# Assessment of radial glia in the frontal lobe of fetuses with Down syndrome

**DOI:** 10.1186/s40478-020-01015-3

**Published:** 2020-08-20

**Authors:** Ana A. Baburamani, Regina T. Vontell, Alena Uus, Maximilian Pietsch, Prachi A. Patkee, Jo Wyatt-Ashmead, Evonne C. Chin-Smith, Veena G. Supramaniam, J. Donald Tournier, Maria Deprez, Mary A. Rutherford

**Affiliations:** 1 Centre for the Developing Brain, School of Biomedical Engineering and Imaging Sciences, King’s College London, London, SE1 7EH UK; 2 University of Miami Brain Endowment Bank, Miami, FL 33136 USA; 3 Department of Biomedical Engineering, School of Biomedical Engineering and Imaging Sciences, King’s College London, London, SE1 7EH UK; 4 Neuropathology and Pediatric-Perinatal Pathology Service [NaPPPS], Holly Springs, MS 38635 USA

**Keywords:** Cortical development, Radial glia, Fetal brain, Down syndrome, SOX2, Diffusion MRI

## Abstract

Down syndrome (DS) occurs with triplication of human chromosome 21 and is associated with deviations in cortical development evidenced by simplified gyral appearance and reduced cortical surface area. Radial glia are neuronal and glial progenitors that also create a scaffolding structure essential for migrating neurons to reach cortical targets and therefore play a critical role in cortical development. The aim of this study was to characterise radial glial expression pattern and morphology in the frontal lobe of the developing human fetal brain with DS and age-matched controls. Secondly, we investigated whether microstructural information from in vivo magnetic resonance imaging (MRI) could reflect histological findings from human brain tissue samples. Immunohistochemistry was performed on paraffin-embedded human post-mortem brain tissue from nine fetuses and neonates with DS (15–39 gestational weeks (GW)) and nine euploid age-matched brains (18–39 GW). Radial glia markers CRYAB, HOPX, SOX2, GFAP and Vimentin were assessed in the Ventricular Zone, Subventricular Zone and Intermediate Zone. In vivo diffusion MRI was used to assess microstructure in these regions in one DS (21 GW) and one control (22 GW) fetal brain. We found a significant reduction in radial glial progenitor SOX2 and subtle deviations in radial glia expression (GFAP and Vimentin) prior to 24 GW in DS. In vivo, fetal MRI demonstrates underlying radial projections consistent with immunohistopathology. Radial glial alterations may contribute to the subsequent simplified gyral patterns and decreased cortical volumes observed in the DS brain. Recent advances in fetal MRI acquisition and analysis could provide non-invasive imaging-based biomarkers of early developmental deviations.

**Electronic supplementary material:**

The online version of this article (10.1186/s40478-020-01015-3) contains supplementary material, which is available to authorized users.

## Introduction

Down syndrome (DS) is the most frequent genetic cause of intellectual disability, with approximately 750 babies born annually in the UK. DS occurs due to partial or complete triplication of human chromosome 21 (HSA21; Trisomy 21). The neurodevelopmental phenotype is variable and associated with cognitive deficits and impairments in speech, motor and language functions. Whilst the neurological phenotype of DS changes over a lifetime, smaller whole brain volumes, predominantly in the cortex and cerebellum, have been observed in early fetal life [19, 45, 55]. Structural magnetic resonance imaging (MRI) studies show that the cortex develops with a simplified gyral appearance (less folded), reduced overall cortical surface area and volume, and abnormal cortical thickness in children and young adults with DS [33, 34]. These volumetric and more specific cortical alterations are prevalent in later-developing regions such as the frontal and temporal lobes [33, 34].

The over-expression of genes on HSA21, their associated pathways in addition to activation of global cellular responses and compensatory mechanisms of genes not on HSA21, are all thought to promote the atypical phenotypes observed in DS [16, 37, 38]. Human post-mortem studies of the fetal and neonatal brain with DS have described decreases in total and neuronal cell numbers, abnormal neuronal maturation, and altered lamination of the cortex which may contribute to the observed reductions in brain volumes [14, 32, 50, 54, 63], as seen on MRI. Development and expansion of the cerebral cortex is a tightly regulated process, beginning with neural precursor cell proliferation, followed by migration to the cortex, allowing for the formation of axonal connections. In fetuses with DS, decreased proliferation and increased cell death have been observed before 21 gestational weeks (GW) [7, 37, 38].

Cortical neurons and glia arise from radial glia progenitors [3, 21, 67]. Radial glia, present from embryogenesis, orchestrate and support cortical development by creating a scaffolding structure that incorporates signalling cues essential for radial and tangential migration of neurons to reach cortical targets [43, 49]. Proliferation is ongoing within the ventricular zone (VZ) and subventricular zone (SVZ) until 28 GW [31, 68]. The earliest born neurons are destined for deeper cortical layers (layer 5–6), and are subsquently followed by production and migration of upper layer neurons. The expansion of these neural progenitors is particularly important in forming gyrencephalic brains, such as the human brain [4, 5, 13, 21, 39, 46]. Any deviations to this tightly regulated process will impact normal neuronal migration, maturation and cortical development.

In the fetal brain with DS, it is not known whether radial glia are morphologically or functionally normal, or whether they contribute to atypical neuronal and cortical development. We have previously shown that in other forms of genetic mutations (*ACTG1* variant), disturbances in radial glia and migratory signals dramatically affect brain development [62]. We and others recently found that quantifiable deviations in cortical growth arise from 28 to 30 GW in fetuses with DS, measured from T2-weighted fetal magnetic resonance imaging (MRI) [45, 55]. Altered radial glial function, impacting neuronal migration may underlie these cortical disturbances. Histological assessments of human post-mortem tissue provide the microscopic resolution to assess radial morphology and recent advances in in vivo MRI now provide the ability to acquire gestation-matched microstructural information about the developing brain.

The aims of this study, therefore, were to characterise radial glia expression pattern and morphology in the frontal lobe in the developing human brain with DS and age-matched euploid controls during the fetal period. It was hypothesised that alterations to radial glial morphology contribute to the altered cortical phenotypes observed in DS. Secondly, to explore whether microstructural information from in vivo MRI could reflect findings from histological assessment of human brain tissue, potentially providing in vivo biomarkers for specific cellular disturbances.

## Materials and methods

Written informed parental consent was acquired in accordance with the National Health Services (NHS) UK guidelines, and study ethical approval was obtained from the National Research Ethics Services (West London), UK (07/H0707/139).

### Cases

Nine post-mortem brains from fetuses/neonates with DS (post-menstrual age (PMA) range 15^+6^–39^+4^ GW, weeks + days, 5 female/4 male) and 9 euploid age-matched brains from control (PMA 18^+0^–39^+2^ GW, 2 female/7 male) fetuses/newborns were used in this study. These cases were obtained from the Perinatal Pathology Department, Imperial College Health Care NHS Trust, and St Thomas’ Hospital London, UK. PMA was calculated by gestational age (at delivery), plus age at death, therefore this was used as a more accurate measure of age. A clinical post-mortem examination was performed by a perinatal pathologist. All brains were assessed macroscopically and microscopically. Euploid age-matched control cases had no known genetic mutations/diagnosis and showed no significant brain pathology. The case details and neuropathological findings are summarised in Table 1.

**Table 1:** Down syndrome (DS) and age-matched euploid control case details. Summary of clinical case information of human post-mortem cases from age-matched Euploid and DS fetuses

Case	Sex	GA at birth (weeks)	Delivery status	Postnatal age	PMA at death (weeks + days)	Birth weight (g)	Congenital heart defects & clinical context	Neuropathology
*Aged-matched euploid control*								
E-1	M	18 + 0	IUD/STILLBIRTH	x	18 + 0	130	Chronic histiocytic intervillostitis	None
E-2	M	22 + 2	TOP	x	22 + 2	402	Hypoplastic left heart syndrome	None
E-3	M	23 + 0	IUD/MISC	x	23 + 0	570	Probable cervical incompetence, congenital pneumonia	None
E-4	M	23 + 2	NND	5 m	23 + 2	531.84	Associated IUGR, oligohydramnios, congestive heart failure	None
E-5	F	24 + 1	NND	4 h 40 m	24 + 1	660	Extreme prematurity, congestive heart failure	None
E-6	M	24 + 2	IUD/STILLBIRTH	x	24 + 2	738	Constricted umbilical cord, congestive heart failure	None
E-7	M	38 + 4	IUD/STILLBIRTH	x	38 + 4	2690	symmetrical IUGR, cord entanglement/obstruction	None
E-8	F	38 + 6	NND	3d	39 + 1	2640	Hypolobated lungs with marked lymphangiectasia and meconium in terminal air spaces	None
E-9	M	39 + 2	IUD/STILLBIRTH	x	39 + 2	3125	Not identified	None
*Down syndrome*								
DS-1	M	15 + 6	TOP	x	15 + 6	76.9	None	None
DS-2	F	18 + 4	TOP	X	18 + 4	178.1	Multiple malformations including: Pulmonary stenosis, membranous VSD	None
DS-3	F	19 + 5	TOP	X	19 + 5	260	None	None
DS-4	M	21 + 1	TOP	X	21 + 1	429.3	Multiple malformations including: peri-membranous VSD	None
DS-5	F	22 + 5	TOP	X	22 + 5	420	None	Focal periventricular haemorrhages
DS-6	F	23 + 4	TOP	X	23 + 4	850	None	None
DS-7	M	37 + 5	NND	1d 14 h 53 m	38 + 0	3980	lung hypoplasia	WM gliosis, perivascular haemorrhages
DS-8	M	38 + 6	NND	11 h 37 m	38 + 6	3630	HIE, myeloproliferative disorder	HIE Grade III
DS-9	F	32 + 4	INFANT DEATH	6wks 6d	39 + 4	2658	Necrotizing Enterocolitis	Patchy WM gliosis

*F* Female, *HIE* hypoxic-ischemic encephalopathy, *IUD* intrauterine death, *IUGR* intrauterine growth restrictions, *M* Male, *NND* neonatal death, *PMA* post-menstrual age, *TOP* termination of pregnancy, *m* minutes, *h* hours, *d* day, *wks* weeks, *VSD* Ventricular septal defect, *WM* white matter. X indicates not applicable. None indicates structurally normal with no overt pathology/injury

### Tissue preparation

**Fig. 1:**
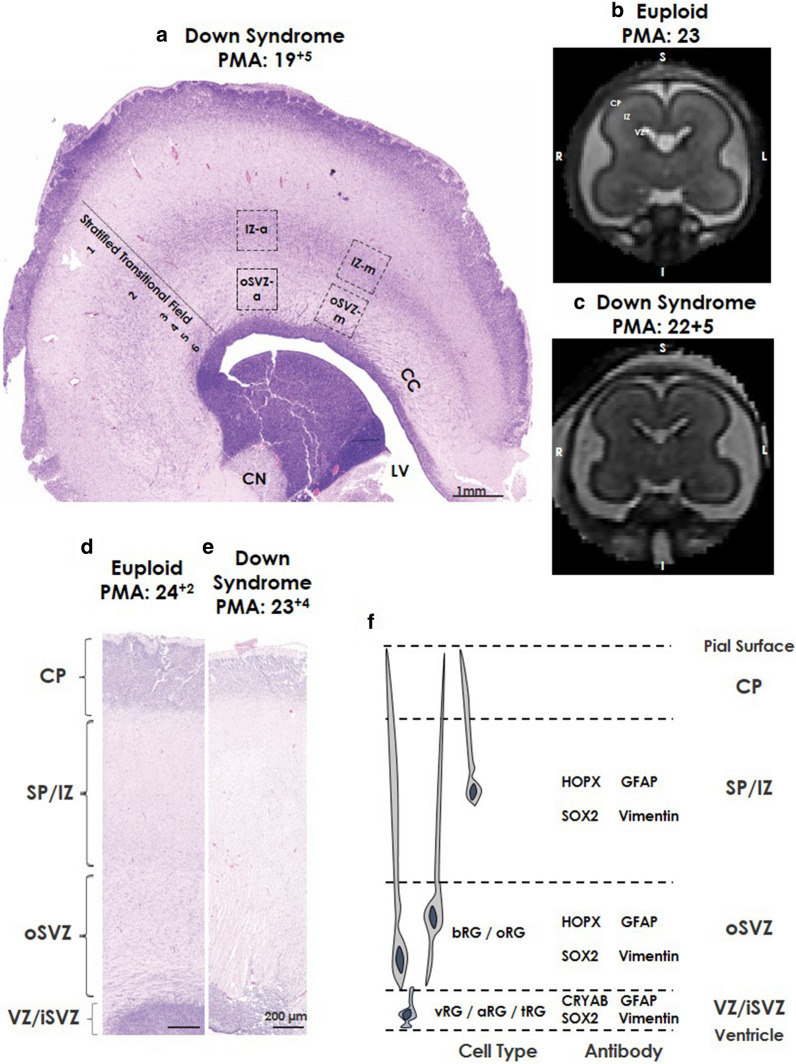
Coronal section of regions of interest. Photomicrographs of Haemotoxlin and Eosin (H&E) stained (**a**) coronal section from the frontal lobe of a Down syndrome case at 19^+5^ weeks PMA showing the stratified transitional field (1–6) and regions of interest. **b**, **c** Coronal T2-weighted MRI images from (**b**) control, 23 weeks PMA and (**c**) Down syndrome, 22^+5^ weeks PMA, highlighting different regions based on T2-weighted signal intensities. Higher magnification of H&E stained frontal lobe coronal sections from (**d**) age-matched Euploid case at 24^+2^ weeks PMA, and (**e**) Down syndrome case at 23^+4^ weeks PMA. **F** Schematic of regions of interest and associated protein labels of radial glia. Scale bars indicated (**a**) 1 mm, (**b**, **c**) 200 µm. Abbreviations: –*a* anterior, *CN* caudate nucleus, *CC* corpus callosum, *CP* cortical plate, *IZ* intermediate zone, *LV* lateral ventricle, -*m* medial, *RG* radial glia (aRG; apical, bRG basal, oRG outer, vRG; ventral, tRG; truncated), *SVZ* subventricular zone (iSVZ; inner, oSVZ; outer), *PMA* post-menstrual age, *STF* stratified transitional field, *SP* subplate, *VZ* ventricular zone

Following post-mortem examination, brains were routinely bisected by a pathologist, sampled from one hemisphere, and fixed with 4% formalin for 5–7 weeks (depending on size). The hemisphere tissue was from (left/right) was not always noted. Paraffin-embedded tissue blocks of the frontal lobe at the level of the caudate (anterior to Ammon’s Horn) were sectioned coronally at 6 µm (Leica RM2245 microtome, Leica Microsystems Ltd.) on superfrost plus slides [53, 61]. A section was used for routine haematoxylin and eosin (H&E) stain to assess for neuropathological changes and was used to identify our regions of interest (ROI; Fig. 1). To ensure that the ROI’s were consistent and comparable between cases, the sections that contained the anatomical landmarks such as the lateral ventricle and the head of the caudate were included (Fig. 1). Adjacent sections were used for immunohistochemical protocols below (1 section per stain).

### Immunohistochemistry

Routine immunohistochemistry was done as previously reported [53, 61, 62]. Primary and secondary antibodies are detailed in Table 2.

**Table 2:** Immunohistochemistry details. Primary and secondary antibody, Avidin–Biotin Complex (ABC) and DAB details [22, 26, 43, 47, 48]

Antigen	Target	Species	Dilution	Cat number	Source
Alpha-crystallin B chain (CRYAB)	Apical (ventral/truncated) radial glia	Anti-mouse	1:250	ab13496	abcam
Homeodomain-only protein (HOPX)	Basal (outer) radial glia	Anti-rabbit	1:250	11419-1-AP	Proteintech
SRY (sex determining region Y)-box 2, (SOX2)	Progenitor cell and radial glial marker	Anti-rabbit	1:500	ab97959	abcam
Vimentin	Intermediate filament protein—Radial glia fibres and cell body	Anti-rabbit	1:500	M072501-2	DAKO
glial fibrillary acidic-protein (GFAP)	Intermediate filament protein—Radial glia fibres and cell body	Anti-rabbit	1:1000	G3893	Sigma
Biotinylated IgG	Secondary antibody	Horse anti-mouse	1:200	BA-2000	Vector Labs
Biotinylated IgG	Secondary antibody	Goat anti-rabbit	1:200	BA-1000	Vector Labs
Avidin-Biotin Complex–Horseradish Peroxidase (ABC-HRP)			1:200	PK-6200	Vector Labs
3,3’-diamino-benzidine (DAB)			1:10	34002	Thermo

### Neuroanatomy and regions of interest

Figure 1 shows ROIs that were characterised based on previously reported protein labels, laminar organization and stratified transitional fields (STF) detailed by Altman and Bayer [1]. The STFs are a large region of the developing brain (from ages 6.5 GW–37 GW), which are split into 6 layers [39, 47, 56]; Fig. 1.

The VZ and SVZ are the highly proliferative zones of the developing brain. From 13 GW, the SVZ is further subdivided into the inner SVZ (iSVZ) and the outer SVZ (oSVZ) [10, 39]. In the VZ, SVZ and intermediate zone (IZ) radial glia progenitors can be identified by SOX2, seen from 8 to 10 GW [21, 52, 56]. In the VZ, SVZ and IZ, radial glia and their fibres can be visualised with intermediate filament proteins vimentin and glial fibrillary acid protein (GFAP) which are expressed from early as 6 GW [6, 11, 22, 67].

VZ/iSVZ: The VZ lines the lateral ventricle and is a high cellular dense area. The iSVZ is also highly cell dense, and contains intermediate progenitors [10, 46]. Both the VZ and iSVZ contain nuclei in an irregular organisation, in contrast to the radial orientation of cells in the oSVZ [21, 51]. Whilst there are molecular differences between the iSVZ and VZ, we have not assessed these and therefore have not discriminated between VZ/iSVZ but were able to visually discriminate the VZ/iSVZ from oSVZ (Fig. 1). In the human VZ/iSVZ, around 16–18 GW, apical radial glia become truncated, terminate in the oSVZ and can be identified with alpha-crystallin B chain (CRYAB) proteins [43, 56].

oSVZ: The oSVZ is directly superior to VZ/iSVZ and comparable to STF 4–6. We assessed the oSVZ-anterior (oSVZ-a) and oSVZ-medial (oSVZ-m) that were anterior to the caudate head [1, 43, 48]. The oSVZ-m was marked at approximately 30º to the caudate head (i.e. above the thickest region of the ganglionic eminence) (Fig. 1). Basal radial glia, present in the oSVZ, can be identified using homeodomain protein (HOPX) [43].

IZ: The IZ lies between the proliferative zones (VZ and SVZ) and the subplate and the cortical plate (CP). It lies superior to the ROIs described for the oSVZ, we assessed IZ-anterior (IZ-a) and IZ-medial (IZ-m), respectively, and comparable to STF 2–3 (Fig. 1).

### Microscopy and image analysis

CRYAB and HOPX staining was visualised and qualitatively assessed with bright-field microscopy (Leica DM6000B, Leica Camera CTR6000, Leica Microsystems Ltd., UK) as previously described [53, 61].

SOX2, GFAP and Vimentin staining was visualised and assessed using Scanning Light Microscope (Motic Easy Scan Pro 6, USA), with extended depth of field capabilities, scanned at 40× magnification resolution. We objectively quantified positively stained particles of SOX2, GFAP and Vimentin, using Image Pro Premier Software, (Media Cybernetics, USA). An intensity threshold was set for each image (30–140 elements), in all four regions (oSVZ-a; oSVZ-m; IZ-a; IZ-m; Fig. 1) from samples that encompassed an ROI area (1.6–3.7 mm^2^). Using a colour analysis, we found that intensity ranges above 140 and below 30 would detect non-specific staining. The number of SOX2-immunopositive cells (minimum SOX2-positive particle area: 5 μm^2^) was calculated. GFAP-immunopositive and Vimentin–immunopositive cells and radial projections that occupied 7 μm^2^ to 1000 μm^2^ were included in analysis, to measure both percentage area stained and number of particles/mm^2^ (inclusive of some cells and radial fibers). Care was taken to exclude staining artifacts and exclude blood vessels (where possible). Additional morphology parameters were also obtained for GFAP and Vimentin-immunopositive particles; particle length (µm), particle area (µm^2^) and circularity (Cir = (4*A)/Pi*MaxFeret^2^; A = area and MaxFeret = maximum diameter, largest distance between two tangents). A circularity value of 0 = linear object, and value of 1 = perfectly circular object. We averaged anterior and medial ROIs to achieve total values for the oSVZ and IZ. Data was normalized to obtain immunopositive particles per mm^2^.

We determined the space between the radial glia processes in the IZ in sections labelled with GFAP and Vimentin. Using Otolith application (Image-Pro Premier; Media Cybernetics, USA) (Additional file 1: Figure S1), the image threshold was set, and 3 evenly spaced lines (approx. 1 mm) were drawn across the ROI (stained blood vessels were removed from the quantification). An intersection was classified as the point where a radial glia process and line meet. The number of intersections for each line and the space between the points of intersection (circuli; μm^2^) was calculated. Data from the 3 lines from IZ-a and IZ-m were then averaged to obtain a value for each case.

### Data analysis

All immunohistopathology was analysed blinded to case. In our mid-gestation (15^+6^–24^+2^ GW) cohort; we qualitatively described the pattern of radial glia staining (CRYAB, HOPX, SOX2, GFAP, Vimentin) in the VZ/iSVZ. In the oSVZ and IZ, we objectively quantified positive staining of SOX2, GFAP and Vimentin. In our late-gestation cases (38^+0^–39^+4^ GW), due to low numbers we visually assessed CRYAB, HOPX, SOX2, GFAP and Vimentin. Statistical analyses were performed on SOX2, GFAP and Vimentin data using IBM SPSS for Windows software package (Version 25, IBM Corp., USA). Data was tested for normality using the Shapiro–Wilk test. Groups were compared using a General Linear Models (GLM) - ANCOVA, with PMA included as a covariate. Significance was set at P < 0.05. Graphs were produced using GraphPad Prism (version 8.0, GraphPad Software, San Diego, CA).

### Fetal MRI patient recruitment

Two fetal cases were investigated to provide age-matched in vivo data on tissue microstructure. Ethical approval for fetal MRI was obtained from the West London and GTAC Research Ethics Committee (07/H0707/105). A control case (22 GW, no congenital abnormalities) and a DS case (21 GW) were recruited from the antenatal clinic at St Thomas’ Hospital. Diagnosis of DS was confirmed at birth.

### Fetal MRI image acquisition

Images were acquired on a 3T MRI System (Philips Achieva; Philips Medical Systems, Best, The Netherlands) using a 32-channel cardiac array coil placed around the maternal abdomen using protocols developed for the developing Human Connectome Project [http://www.developingconnectome.org/] [12, 45]. Fetal T2-weighted and diffusion tensor imaging was acquired as previously detailed [9, 45].

### Fetal diffusion processing and analysis

To explore a potential role for in vivo fetal MRI to define imaging correlates for underlying biological substrates, we assessed diffusion MRI-derived fibre directions in the fetal brain. Diffusion MRI is sensitive to microstructural properties in the order of cellular length scales and provides 3D spatial and angular information.

The pre-processing for diffusion MRI volumes included image denoising based on random matrix theory [8] and distortion correction [9]. The top shell (b = 1000 s/mm^2^) was used for motion correction and reconstruction of the diffusion weighted signal in the 4th order Spherical Harmonic basis using the method developed by [12] to a resolution of 2 × 2 × 2 mm^3^. This was followed by estimation of fibre orientation distributions (FODs) using white matter constrained spherical deconvolution [58]. The FODs were used as an input for probabilistic tractography [57] (performed in MRtrix3 [59]) in the segmented brain white matter region. Contrary to the common usage of tractography which aims to extract long-range connectivity between brain regions, we extracted local information that approaches the length-scale of the histological samples. Hence, to link local patterns across voxels, we performed tracking with comparatively short minimum streamline length (3 mm) and truncated streamlines at 15 mm.

In both the control and DS case, the streamlines were qualitatively analysed for 5 voxel-sized ROIs, selected to approximately correspond to the ROIs in the post-mortem histology samples (oSVZ-m, oSVZ-a, IZ-m, IZ-a) and the centre of the corpus callosum.

## Results

Cases were divided into mid-gestation (Euploid; n = 6, PMA range: 18^+0^–24^+1^ GW, DS; n = 6, PMA range: 15^+6^– 23^+4^ GW) and late gestation (Euploid n = 3, PMA Range: 38^+4^ –39^+2^ GW; DS n = 3, PMA Range: 38^+0^–39^+4^ GW). One euploid and two DS cases had a congenital heart defect, with no overt brain injury or malformation. The euploid controls and five of the DS cases did not show any brain injury or malformation on gross and microscopic examination, as assessed by a pathologist. Two DS cases had evidence of white matter gliosis (both died postnatally), one had hypoxic-ischemic encephalopathy (HIE) (died < 12 h of birth), and two had evidence of peri-vascular/ventricular haemorrhages (posterior to regions assessed in this study). All case details are listed in Table 1. Based on H&E staining, DS cases from 20 GW (3/3 cases; Fig. 1e) showed that the CP and the VZ had a sparse cellular pattern when compared to the euploid controls (Fig. 1d).

### Expression of CRYAB and HOPX across gestation

In the VZ/iSVZ and oSVZ we visually assessed radial glia markers CRYAB and HOPX at mid-gestation (PMA age range: 15–24 GW), and late gestation (38–39 GW), (Tables 1, 3).

**Table 3:** CRYAB and HOPX staining in the Ventricular and Subventricular Zones during mid-gestation in Down syndrome and age-matched euploid control brains

Cases	PMA (weeks + days)	CRYAB		HOPX	
		VZ/iSVZ	oSVZ	VZ/iSVZ	oSVZ
*Aged-match euploid control*					
E-1	18 + 0	Peri-nuclear, along ventricle	X	Sparse	Yes
E-2	22 + 2	Peri-nuclear, heterogeneous expression	continuous radial projection	Sparse	Yes
E-3	23 + 0	Peri-nuclear, along ventricle	X	Sparse	Yes
E-4	23 + 2	Peri-nuclear, along ventricle	Punctate radial projections	Sparse	Yes
E-5	24 + 1	Strong radial, away from ventricle - perinuclear, tails to positive cells	X	Sparse	Yes
*Down syndrome*					
DS-1	15 + 6	X	X	Sparse	Yes
DS-2	18 + 4	Peri-nuclear and radial	X	Sparse	Yes
DS-5	19 + 5	Peri-nuclear along ventricle	Punctate radial projections	Sparse	Yes
DS-4	21 + 1	Cellular, peri-nuclear and radial	Punctate radial projections	Sparse	Yes
DS-5	22 + 5	X	X	Sparse	Yes
DS-6	23 + 4	X	X	Sparse	Yes

*E* euploid, *DS* down syndrome, *PMA* post-menstrual age, *VZ* ventricular zone, *iSVZ* inner subventricular zone, *oSVZ* subventricular zone. X = no positive staining present, Sparse = few cells; yes = frequent number of cells

**Fig. 2:**
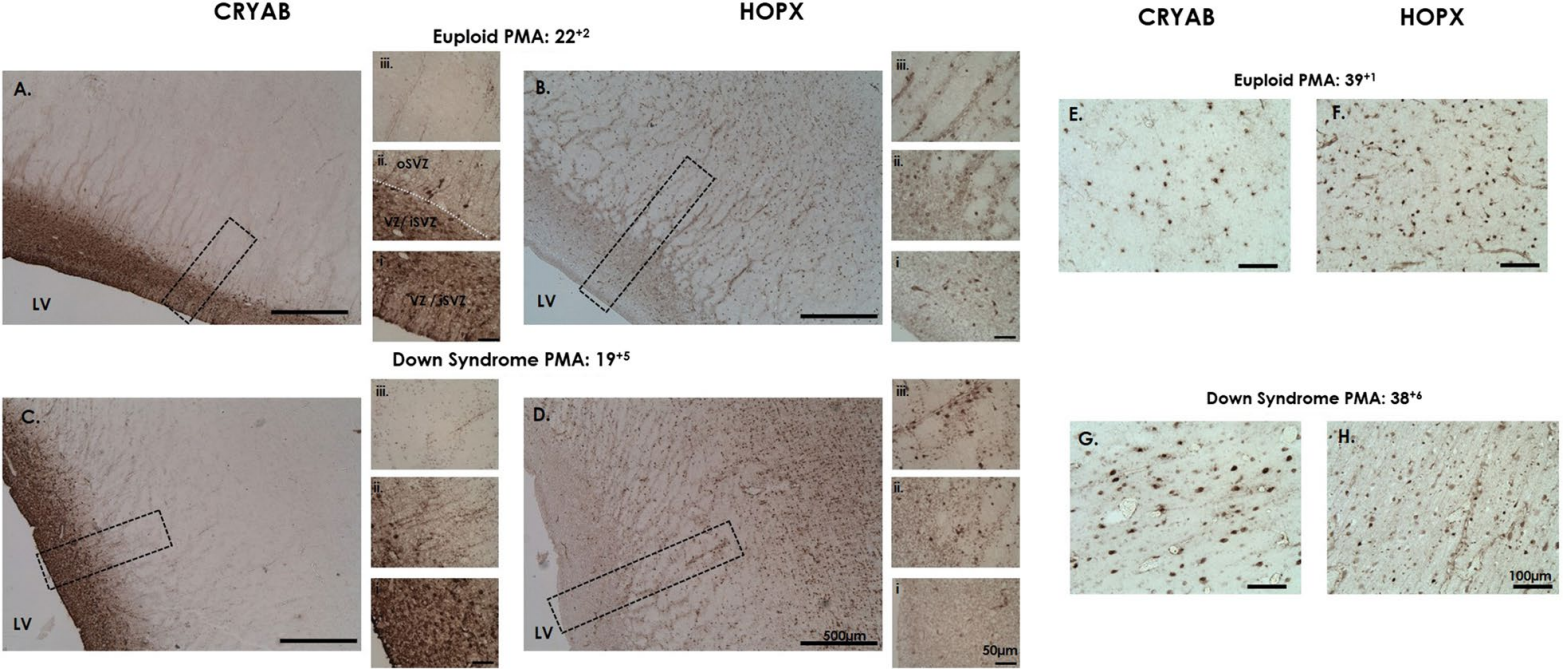
CRYAB and HOPX in the developing brain. Photomicrographs of CRYAB (apical/ventral/truncated radial glia), and HOPX (basal/outer radial glia) in coronal frontal lobe sections in mid-gestation from (**A**, **B**) age-matched euploid, 22^+2^ weeks PMA and (**C**, **D**) Down syndrome 19^+5^ weeks PMA. (**E**–**H**) In late-gestation CRYAB and HOPX staining is cellular in both Down syndrome (38^+4^ weeks PMA) and euploid (39^+1^ weeks PMA) cases. Scale bars indicate (**A**–**D**) 500 µm, (i–iii) 50 µm, (**E**–**H**) 100 µm. Abbreviations: LV; lateral ventricle, SVZ; subventricular zone (iSVZ; inner, oSVZ; outer), PMA; post-menstrual age, VZ; ventricular zone

*CRYAB* antibody stains apical (ventral/truncated) radial glia (Fig. 1f); [43, 56]. We found no CRYAB staining at 15 GW. From 18 to 24 GW CRYAB was present in cells and radial processes in the VZ/iSVZ (Table 3; Fig. 2A, C) in both DS and euploid cases. The radial projections were either punctate (3 cases) or continuous (1 case). No CRYAB staining was found in the IZ. In late gestation, CRYAB staining was localised predominantly to cells of astroglial morphology in the VZ and were now detected in white matter (former IZ) in both DS (Fig. 2G) and euploid cases (Fig. 2E).

HOPX stained basal radial glia (Fig. 1f), and was present in the nucleus and present in a proportion of cell processes [27, 43, 48]. From mid-gestation (15–24 GW), in both DS and euploid cases there was sparse expression of HOPX present in the VZ/iSVZ (Table 3; Fig. 2Bi, ii and Di, ii), strong expression in the oSVZ (Fig. 2Biii and Diii), and sparse expression in the IZ. In late gestation, the HOPX staining was nuclear, with a proportion of cells also having HOPX-positive processes. These were detected across all layers from the VZ, through to the cortex of both DS (n = 3, Fig. 2H) and euploid brains (n = 3, Fig. 2F).

### SOX2 cells are decreased in the oSVZ in the DS brain

**Fig. 3:**
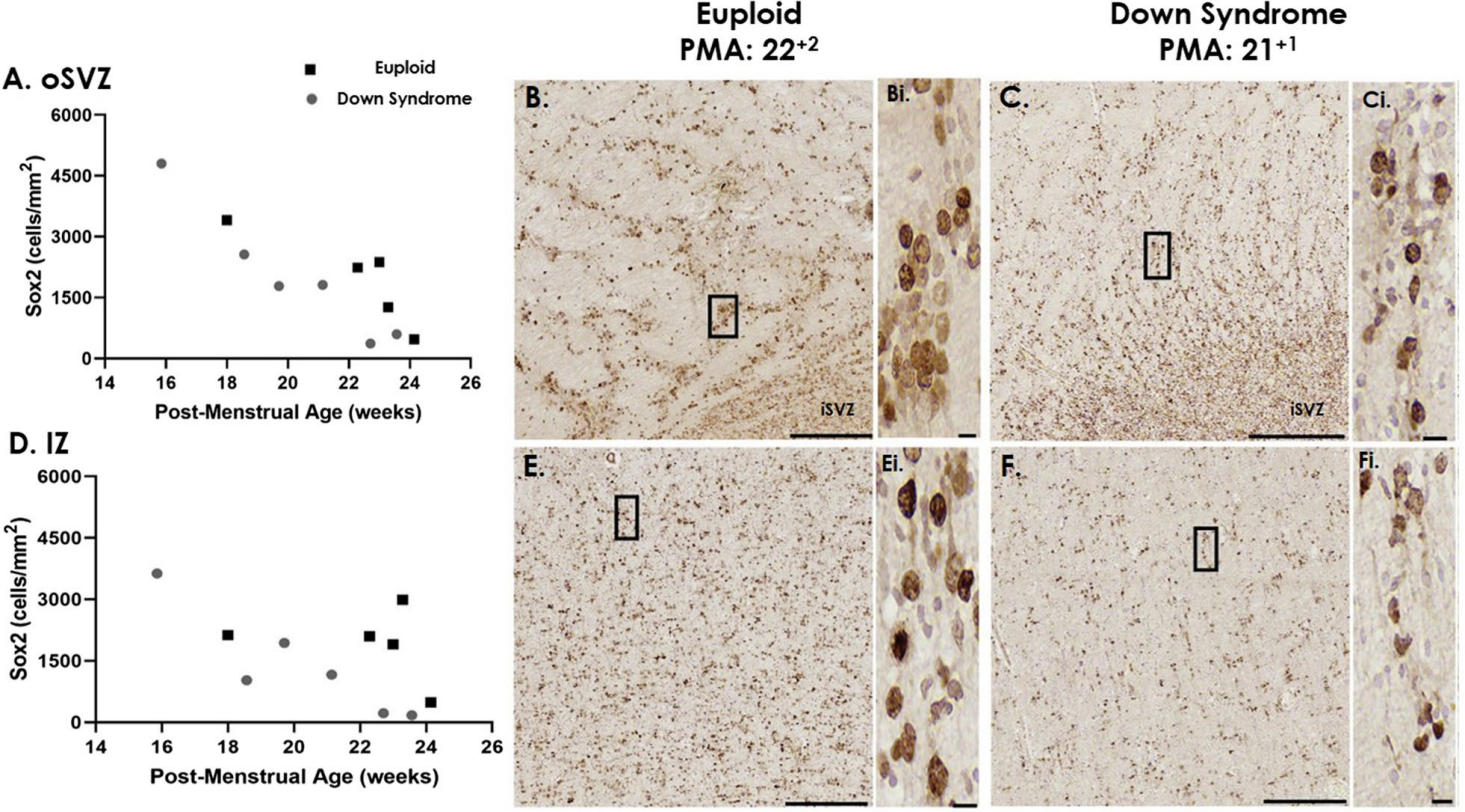
SOX2 cells in the oSVZ and IZ in mid-gestation. The number of SOX2 positive cells in the (**A**) oSVZ and (**D**) IZ across mid-gestation in age-matched euploid (black squares) and Down syndrome (grey circles) cases. Photomicrographs of SOX2 staining in the (**B, C**) iSVZ/oSVZ and (**E, F**) IZ in (**B, E**) euploid, 22^+2^ weeks PMA and Down syndrome, 21^+1^ weeks PMA. Scale bars indicate (**B, C, E, F**) 150 µm, (i) 5 µm. Abbreviations: IZ; intermediate zone, SVZ; subventricular zone (iSVZ; inner, oSVZ; outer), PMA; post-menstrual age

SOX2 is a neural stem progenitor cell marker (expressed in neural progenitors and populations of intermediate progenitors) and is highly expressed in radial glia cells [21, 24, 40, 43, 48, 56]. From mid-gestation (15–24 GW), in both euploid and DS brains (Fig. 3B, C), SOX2 was strongly expressed in the VZ/iSVZ but appeared cell dense in the euploid (Fig. 3B, E) compared to DS cases (Fig. 3C, F). In the oSVZ, SOX2 positive cells were present along radial striations (Fig. 3B, C) in both euploid and DS cases.

The number of SOX2 positive cells was quantified in the oSVZ and IZ from mid-gestation (Fig. 3). We found cellular expression was highest early in development, and decreased across gestation in both euploid and DS frontal lobe sections. The number of SOX2 positive cells were greater in the oSVZ compared to the IZ. We found a significant difference between DS and euploid groups for SOX2 cells in the oSVZ (GLM, F = 6.173, p < 0.05). There was a trend that SOX2 cell number was lower in DS brains, at comparable gestational ages in the IZ (GLM, F = 4.118, p = 0.077). In late gestation, both euploid and DS, SOX2 immunopositive cells were sparse throughout both the euploid and DS brain.

### GFAP and vimentin in DS and euploid frontal lobe from 15 GW show subtle deviations in the DS brain

GFAP and Vimentin are intermediate filament proteins expressed in radial glia as early as 5–6 GW [11, 22, 26, 67]. We saw strong GFAP and Vimentin positive staining present throughout the DS and euploid brain, from 15 GW, in the VZ through to the CP. At these ages (15–24 GW), both GFAP and Vimentin-positive staining was present in radial fibres and in some cell bodies. Vimentin positive staining was also present in blood vessels but these structures were omitted from the radial glia quantification.

**Fig. 4:**
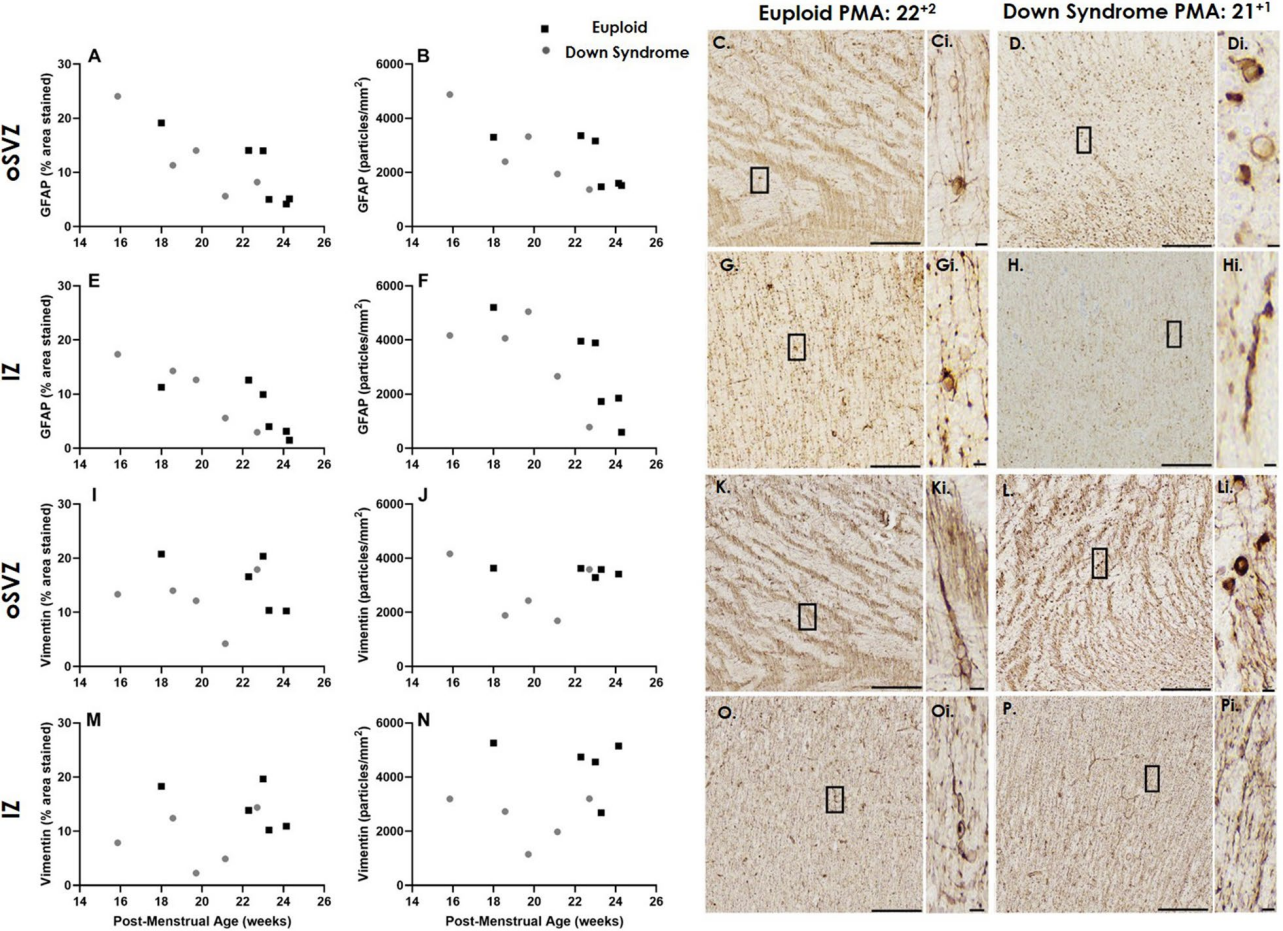
Amount and Number of GFAP and Vimentin in the oSVZ and IZ during mid-gestation. The amount (% area stained) and number (particles/mm^2^) of (**A, B, E, F**) GFAP and (**I, J, M, N**) Vimentin in the (**A, B, I, J**) oSVZ and (**E, F, M, N**) IZ during mid-gestation in age-matched euploid (black squares) and Down syndrome (grey circles) cases. Photomicrographs of (**C, D, G, H**) GFAP and (**K, L, O, P**) Vimentin in the (**C, D, K, L**) oSVZ and (**G, H, O, P**) IZ, in (**C, G, k, O**) Euploid and (**D, H, L, P**) Down syndrome. *IZ* intermediate zone, *oSVZ* outer subventricular zone, *PMA* post-menstrual age. Scale bars indicate (**C, D, G, H, K, L, O, P**) 150 µm, (i) 5 µm

We analysed the amount (% area staining) and number (particles/mm^2^) of GFAP in the oSVZ (Fig. 4A–D) and IZ (Fig. 4E–H) and found that with increasing gestation, GFAP decreased in both euploid and DS brains. In GFAP-positively stained particles (inclusive of radial fibres and some cells), we also found circularity (0 = line/fibres, 1 = circle/cell bodies) had a subtle increase with advancing gestation in both regions in the euploid brain; this increase was not observed in DS (Table 4). No significant differences in percent stained, particle circularity, length and area, between groups, from 15 to 24 GW was observed.

**Table 4:** Morphology parameters of GFAP and Vimentin labelled radial glia in the Down syndrome and age-matched Euploid brain

Cases	PMA	GFAP						Vimentin					
		oSVZ			IZ			oSVZ			IZ		
		Circularity	Area (µm^2^)	Length (µm)	Circularity	Area (µm^2^)	Length (µm)	Circularity	Area (µm^2^)	Length (µm)	Circularity	Area (µm^2^)	Length (µm)
*Age-matched euploid control*													
E-1	18 + 0	0.25	57.98	14.14	0.26	21.64	9.32	0.24	57.13	14.04	0.28	38.65	11.38
E-2	22 + 2	0.22	47.14	14.14	0.23	31.82	12.19	0.23	45.79	13.59	0.25	29.50	11.04
E-3	23 + 0	0.20	44.31	15.20	0.16	25.17	13.80	0.20	61.98	17.16	0.21	40.24	14.62
E-4	23 + 2	0.30	34.43	10.71	0.33	23.14	8.69	0.32	28.86	9.55	0.30	38.10	10.93
E-5	24 + 1	0.35	26.08	8.65	0.43	16.99	6.34	0.34	30.07	9.22	0.33	20.67	8.17
E-6	24 + 2	0.38	31.71	9.12	0.37	22.57	8.09	x	x	x	x	x	x
*Down syndrome*													
DS-1	15 + 6	0.31	49.33	12.44	0.28	42.60	11.40	0.33	32.23	10.17	0.33	24.64	8.97
DS-2	18 + 4	0.22	47.21	14.48	0.20	34.91	12.85	0.20	75.37	19.35	0.16	48.23	17.78
DS-3	19 + 5	0.31	42.52	11.81	0.33	24.87	9.54	0.27	49.87	13.82	0.32	19.80	8.36
DS-4	21 + 1	0.27	29.35	10.17	0.30	21.56	8.65	0.45	24.86	7.59	0.42	24.88	7.93
DS-5	22 + 5	0.29	60.14	13.30	0.27	37.66	13.88	0.32	49.83	13.39	0.33	47.42	13.01

Length, Area and Circularity measured in the oSVZ and IZ across mid-gestation. X; Not assessed

The amount of Vimentin (% area staining) and the number (particles/mm^2^) in the oSVZ (Fig. 4I–L) and IZ (Fig. 4M–P) showed more variability across gestation in both euploid and DS brains. Circularity in the euploid brain regions, showed a subtle increase across gestation, which was not observed in DS brains (Table 4). No significant differences in percent stained, particle circularity, length and area, between groups, from 15 to 24 GW was observed.

**Fig. 5:**
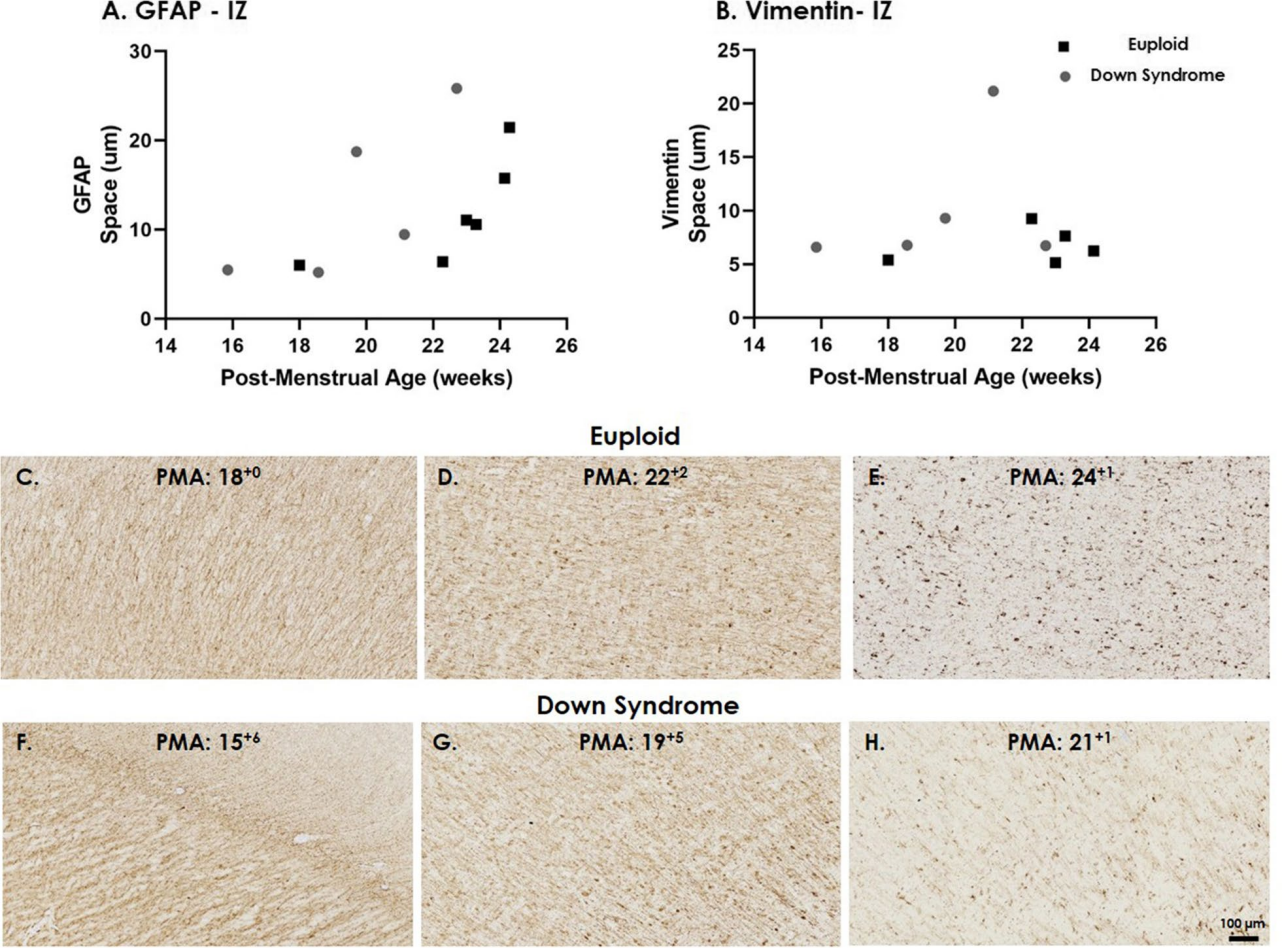
Space between radial glial fibres in the IZ. The average space between (**A**) GFAP and (**B**) Vimentin labelled radial glial fibres in the IZ across mid-gestation in age-matched euploid (black squares) and Down syndrome (grey circles) cases. Photomicrographs of GFAP staining across mid-gestation in (**C–E**) euploid and (**F–H**) Down syndrome cases. Scale bars indicate (**C–H**) 100 µm. *IZ* intermediate zone, *PMA* post-menstrual age

In order to gain further insight into developmental changes that occur in radial glial fibres, we objectively assessed space between fibres in the IZ for GFAP and Vimentin positive radial glia (Fig. 5). The amount of space between GFAP-labelled radial glia appeared to increase across gestation (15–24 GW) in both euploid and DS brains (Fig. 5C–H; Table 4). Vimentin-radial glia showed some variability and there was no increase in average space between fibres across gestation in the IZ.

Due to low number of cases in late gestation, we visually assessed the expression of GFAP and Vimentin from the VZ through to the cortex. GFAP was now present in cells of astrocytic morphology and a small proportion of radial like projections in the cortex (Additional file 2: Figure S2). GFAP-positive astrocytes in one DS case that had HIE, also had evidence of hypertrophic astrocytes (Additional file 2: Figure S2C). Vimentin-positive radial processes were still evident (39 GW), although fewer than earlier in development, in white matter regions in both DS (Additional file 2: Figure S2D) and euploid brains (Additional file 2: Figure S2B). Of interest, Vimentin staining was also present in cells and blood vessels across all gestational ages.

### In vivo diffusion MRI in the fetal brain is comparable to histological assessments

**Fig. 6:**
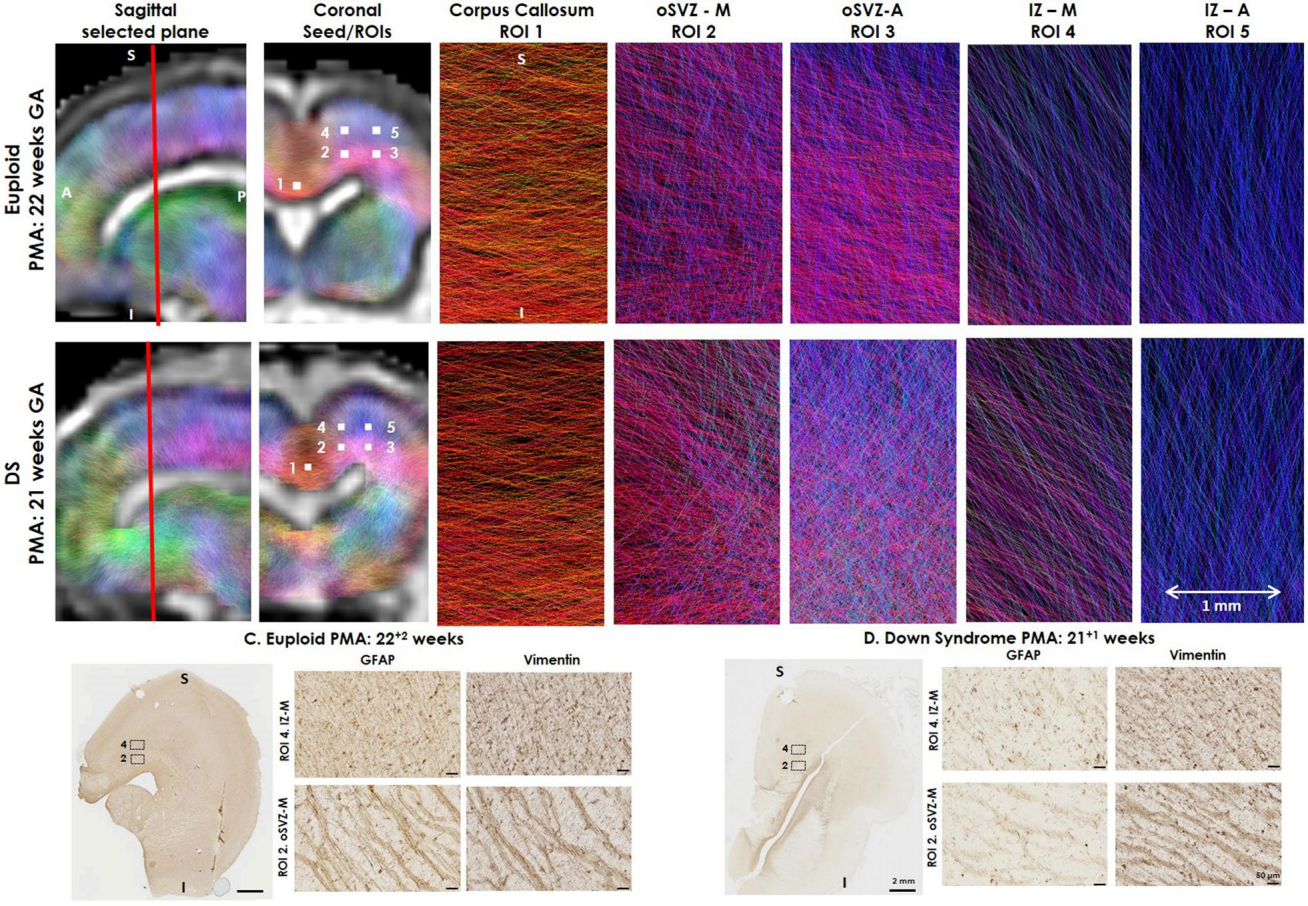
Fetal diffusion MRI and immunohistochemistry in the fetal brain. Upper panels show a (**A**) Euploid (22 GW) and (**B**) DS (21 GW) fetal brain, following in vivo fetal MRI in sagittal plane, and 5 seed/regions of interest (ROI), approximately 1 × 1 × 1 voxel, (2 mm), corresponding to immunohistological ROIs assessed and the corpus callosum. The colour-coding of the tractography is based on the standard red, green, blue code to indicate directionality; red for left–right, green for anterior–posterior and blue for dorsal–ventral. The lower panel reflect GFAP and Vimentin labelled radial glia, with images rotated in a comparable plane to the fetal MRI images in the (**C**) Euploid and (**D**) DS brain, showing direction of fibres in comparable ROI 2 (oSVZ-M) and ROI 4 (IZ-M) stained with GFAP and Vimentin. *S* superior, *I* inferior. Scale bar indicates 2 mm on low magnification and 50 µm on high magnification photomicrographs

Conventional T2-weighted images of both cases, euploid control (22 GW) and DS case (21 GW), were visually assessed by a neuroradiologist and confirmed to have normal appearance, with no injury or malformations and 2D biometry values within normal limits. We assessed in vivo diffusion MRI-derived fibre directions, in selected voxels (2 × 2 × 2 mm^3^) in the fetal brain (Fig. 6). Approximate mean diffusivity (scalar diffusivity) values for the control fetus (n = 1); Ventricle: 2.4 × 10^−3^ mm^2^/s; VZ: 1.1 × 10^−3^ mm^2^/s; SVZ: 1.5 × 10^−3^ mm^2^/s; Subplate/IZ: 1.8 × 10^−3^ mm^2^/s; Cortex: 1.3 × 10^−3^mm^2^/s; Corpus Callosum: 1.0 × 10^−3^ mm^2^/s. In the corpus callosum (ROI1) we observed a strong left to right orientation, as expected. In ROI2 and ROI3 which corresponds to the oSVZ, diffusion MRI revealed a large proportion of streamlines orientated superior to inferior, but a smaller number of streamlines orientated anterior to posterior. This was consistent with our immunohistochemical observations in the oSVZ, where we show strong bands/striations predominantly in one direction, with fewer streamlines distributed in the perpendicular direction (Fig. 6C, D). In the IZ (ROI4, ROI5), we noted that the orientation of streamlines reflected a predominantly radial orientation, consistent with our histological assessments. Importantly, we noted that streamline orientation was different between the oSVZ and IZ regions, on both control and DS brain imaging, consistent with histology.

## Discussion

The aim of this study was to assess radial glial morphology and pattern of expression in DS and euploid age-matched brains during the fetal period. We assessed the VZ/iSVZ, oSVZ and IZ from mid-gestation 15–24 GW, which are regions that are structurally important for the generation and migration of neurons during this developmental window. We found the pattern of expression of radial glia markers was comparable, with subtle deviations between age-matched euploid and DS fetal brains. SOX2 cell numbers were significantly lower in the oSVZ in DS compared to euploid brains. Although numbers of cases assessed were small, with advancing gestation (15 to 24 GW), we found SOX2 cell number and amount and number of GFAP particles to decrease in both the oSVZ and IZ, and this occurred earlier in the DS brains compared to controls. There was no strong relationship with Vimentin across gestation in either region or group. We also show that recent advances in in vivo fetal diffusion MRI could complement and reflect histological analysis and allow for insight into the microstructural development from as early as 21 GW, potentially providing in vivo biomarkers of development.

We found a significant difference in the number of SOX2 cells in the oSVZ of DS brains, from 15 to 24 GW compared with age-matched euploid cases (Fig. 3). Due to the small number of cases assessed and the individual variability that exists in DS, this finding is inconclusive. However, it is consistent with several studies that have found reduced proliferation in the VZ and SVZ of DS brains, prior to 21 GW [7, 17, 18]. In the non-DS, typically developing brain, it has been reported that from 13 to 18 GW (earlier than we assessed), SOX2 cell numbers decrease in the VZ, and increase in the oSVZ suggesting this is when the oSVZ becomes the predominant proliferative zone [21, 35, 43, 48]. Basal radial glia populate the oSVZ, and from 17 to 18 GW compared to 23–24 GW there is a shift in the production of deep layer neurons to upper layer neurons [43]. Thus a reduction in SOX2 in the DS oSVZ brain may reflect decreased neuronal numbers and lack of cellular layer definition that has been previously described in layers II, III and IV in the developing DS cortex [14, 50, 54, 63, 64]. We also found SOX2 cell number decreased with advancing gestation in the oSVZ and IZ in both DS and euploid brains, with lower numbers in the DS brain. SOX2 protein is essential to ensure self-renewal of neuronal precursors and its expression is downregulated following differentiation to post-mitotic neuronal and glial cells [15, 20]. In late gestation, there were few SOX2 cells in either the DS or euploid brain, in agreement with Malik et al., who found after 28 GW SOX2 cells were more scarce in the SVZ (in the non-DS brain) [40].

We assessed the spatio-temporal pattern of apical (CRYAB) and basal (HOPX) radial glia and found it to be comparable in our DS and euploid brains. We found CRYAB in the VZ/iSVZ from 18 GW in both euploid and DS brains, with CRYAB-fibres truncating in the oSVZ, consistent with previous studies reporting the emergence of ‘discontinuous’ radial glia from 16 to 18 GW [11, 43, 48, 56]. In mid-gestation the morphology of CRYAB staining was consistent with GFAP and Vimentin positive fibres in the VZ and in late-gestation, CRYAB staining was cellular, with star shaped morphology consistent with astroglia in the VZ and white matter. CRYAB has been shown to co-localise with GFAP-positive astrocytes and PDGFRa (a marker of oligodendrocyte progenitors) in DS and control infants [30, 44]. Basal radial glia, labelled with HOPX, were sparse in the VZ/iSVZ, and strongly expressed in the oSVZ from 15 GW in both DS and euploid, with some HOPX cells containing short processes. Recently, it was found that at 15 GW, proliferating basal radial glia, labelled with SOX2 (nucleus) and HOPX (nucleus and processes) contained 6 distinct morphologies, with an increase in cellular complexity correlating with increased proliferation [27]. Whilst we did not analyse the number or morphology of our HOPX positive-cells, it would be of interest to further assess the relationship between HOPX cellular morphology and proliferation in the DS brain.

Once born, neurons begin a multi-step process to migrate along radial glial fibres from their birthplace, through the IZ and subplate to their final cortical location. Radial, tangential and multi-polar migration all occur with the support of radial glia fibres. We found the number and the amount of GFAP and Vimentin-positive staining decreased with advancing gestation, with this occurring earlier in DS brains compared to age-matched euploid brains. Cortical neurogenesis continues till at least 28 GW [40], and following peak neuronal production, radial glial cells begin to switch to gliogenesis. The slightly shifted pattern of expression of GFAP and Vimentin (and SOX2) in DS brains may reflect radial glia maturing into astroglia earlier, or becoming exhausted sooner which has been previously noted in the VZ of foetuses with DS between 18–20 GW [66]. In the VZ of DS fetuses (< 20GW) a greater number of GFAP-positive cells and vimentin-labelled radial glia was reported [37, 38, 66]. Guidi et al., (2018) have reported a comparable number of GFAP-labelled astrocytes and reduced number of neurons, suggestive of increased percentage of astrocytic cells (< 21GW), in the fusiform and inferior temporal gyrus [18]. We did not assess neuronal number in this study, but the decreased number of SOX2 cells in the oSVZ of DS and observed decreases in cortical volumes from fetal MRI studies could reflect decreased neuronal numbers observed in the cortex of the DS brain after birth [14, 45, 50, 55, 63].

In the IZ, we also assessed the space between GFAP-labelled fibres and found that this increased with advancing gestation in both the DS and euploid brain. Less densely packed radial glia have been reported from 24 to 29 GW, when compared to earlier in development 19–21 GW [65]. This maturational process could reflect radial fibres transitioning into mature astrocytes, which occurs between 15 and 35 GW [11, 41, 65, 67]. Space between vimentin fibres remained fairly consistent across 15–24 GW in our study comparable with other studies that have documented vimentin stained radial glia only regressing from 30 GW [11, 65]. Whilst GFAP and vimentin have a high co-expression, subtle differences in the pattern of expression have been previously reported [6, 11, 22, 67]. Perturbations to extracellular matrix components, which occupy the space between glia and neurons, have recently been shown to be involved in cortical folding, and are altered in the DS brain [36]. The extracellular matrix follow a tightly regulated process that supports the development, maintenance and organisation of the brain [25], our ongoing histological and MRI studies will further explore its relationship with GFAP and vimentin-labelled radial fibres.

Our preliminary assessment of fetal diffusion MRI from one DS and control fetal case showed that as early as 21 GW, it is possible to visualise streamlines reflecting radial fibre orientations in vivo in the developing fetal brain. Importantly, our immunohistochemistry and diffusion MRI showed a different pattern of radial glial fibre orientation between the oSVZ and IZ, in both the DS and age-matched euploid brains during mid-gestation. The oSVZ had bands of striations, with prominent spaces between these bands, whilst the IZ had traditional radially orientated fibres (Fig. 6). Correlating with histological studies, diffusion MRI of human post-mortem brains has added valuable insight into the development, pattern and regression of radial tracts across gestation [23, 60, 65]. From 30 to 41 GW radial glia have been documented to disappear [29, 65], with radial migration pathways beginning to regress as early as 22 GW in the inferior frontal cortex [42]. While our example demonstrates that fetal diffusion MRI and histology detect features with similar microstructural orientation, future research will require extensive parametric study for both reconstruction and analysis methods as well as an assessment of inter-subject variability to explore the development of the fetal brain in DS more quantitatively across gestation.

Whilst access to and assessment of human post-mortem tissue is immensely valuable, it has some limitations. The number of cases and amount of tissue available is limited. The presence of other pathologies such as acquired injuries or infection, could have further affected the results. The time till post-mortem, process and duration of tissue fixation and paraffin processing can effect tissue integrity and shrink brain tissue in variable degrees which can effect tissue quality and subsequent measures. Due to the low number of cases we chose not to calculate group averages and instead assessed and presented our data across gestation. Although this did limit statistical analysis, it highlighted valuable information regarding developmental changes in protein expression of radial glia, during this active period of neurodevelopment. In addition, it has been recently established that within the DS population, there is large individual variability [2, 28]. We have previously observed a wide range in the total and regional brain volumes in our fetal and neonatal DS brains [45], and such genetic variation needs to be recognised when investigating neurodevelopmental phenotypes in DS. The presence of co-morbidities such as a congenital heart defect, known to influence brain development in both the DS and non-DS population, may further contribute to individual variations in datasets.

## Conclusions

Our neuropathological and fetal MRI findings show subtle but significant deviations in the DS brain prior to 24 GW, consistent with previous studies [50, 63]. We found SOX2 cell number was significantly less in DS, and whilst both DS and controls have a similar pattern of expression, these occur earlier in DS. These alterations could further contribute to the simplified gyral patterns and decreased cortical volumes that emerge in the DS brain. Recent advances in fetal MRI acquisition and analysis techniques taken together with a comparison with detailed regional histology could help define underlying biological substrates for objective MR imaging parameters. This now provides the potential for in vivo, non-invasive imaging based surrogate markers that could predict subsequent alterations in cortical development and their later neurocognitive correlates.

## Supplementary information

**Additional file 2: Figure S1.** Schematic of Otolith Application. To measure the distance between radial glial fibres the Otolith Application was used. In each of the images assessed, three 1000 µm transect lines (red line) were drawn at different points on the edge of each scan. The outer reference circle was formed at the endpoint of each transect line (blue arrow). The application identified the circuli or radial structures (µm). The number of circulii and the distances between each radial projection was calculated and this data was analysed. Scale bar = 30 µm.

**Additional file 2: Figure S2.** GFAP and Vimentin in late gestation. (A, C) GFAP and (B, D) Vimentin in the late gestation brain in (A, B) euploid age-matched (38^+4^ weeks PMA) and (C, D) DS (38^+6^ weeks PMA). DS case had Hypoxic-Ischemic Encephalopathy, with evidence of reactive astrocytes (C). Scale bar indicate 100 µm.

## Data Availability

The data that support the findings of this study are available from the corresponding author upon reasonable request.

## Abbreviations

DS: Down syndrome
GW: Gestational weeks
GFAP: Glial fibrillary acid protein
HSA21: Human chromosome 21
IZ: Intermediate zone
MRI: Magnetic resonance imaging
PMA: Post-menstrual age
ROI: Region of interest
SVZ: Subventricular zone
VZ: Ventricular zone

## References

[1] Altman J, Bayer SA (2002). Regional differences in the stratified transitional field and the honeycomb matrix of the developing human cerebral cortex. J Neurocytol.

[2] Baburamani AA, Patkee PA, Arichi T, Rutherford MA (2019). New approaches to studying early brain development in Down syndrome. Dev Med Child Neurol.

[3] Beattie R, Hippenmeyer S (2017). Mechanisms of radial glia progenitor cell lineage progression. FEBS Lett.

[4] Borrell V (2019). Recent advances in understanding neocortical development. F1000 Research.

[5] Borrell V, Reillo I (2012). Emerging roles of neural stem cells in cerebral cortex development and evolution. Dev Neurobiol.

[6] Bramanti V, Tomassoni D, Avitabile M, Amenta F, Avola R (2010). Biomarkers of glial cell proliferation and differentiation in culture. Front Biosci.

[7] Contestabile A, Fila T, Ceccarelli C, Bonasoni P, Bonapace L, Santini D, Bartesaghi R, Ciani E (2007). Cell cycle alteration and decreased cell proliferation in the hippocampal dentate gyrus and in the neocortical germinal matrix of fetuses with Down syndrome and in Ts65Dn mice. Hippocampus.

[8] Cordero-Grande L, Christiaens D, Hutter J, Price AN, Hajnal JV (2019). Complex diffusion-weighted image estimation via matrix recovery under general noise models. NeuroImage.

[9] Cordero-Grande L, Price A, Ferrazzi G, Hutter J, Christiaens D, Hughes E, Hajnal JV (2018) Spin And Field Echo (SAFE) dynamic field correction in 3T fetal EPI. In: Proceedings of the 26th annual meeting of ISMRM, City, pp 208

[10] De Juan Romero C, Borrell V (2015). Coevolution of radial glial cells and the cerebral cortex. Glia.

[11] deAzevedo LC, Fallet C, Moura-Neto V, Daumas-Duport C, Hedin-Pereira C, Lent R (2003). Cortical radial glial cells in human fetuses: depth-correlated transformation into astrocytes. J Neurobiol.

[12] Deprez M, Price A, Christiaens D, Estrin GL, Cordero-Grande L, Hutter J, Daducci A, Tournier JD, Rutherford M, Counsell SJ (2019). Higher order spherical harmonics reconstruction of fetal diffusion MRI with intensity correction. IEEE Trans Med Imaging.

[13] Florio M, Huttner WB (2014). Neural progenitors, neurogenesis and the evolution of the neocortex. Development.

[14] Golden JA, Hyman BT (1994). Development of the superior temporal neocortex is anomalous in trisomy 21. J Neuropathol Exp Neurol.

[15] Graham V, Khudyakov J, Ellis P, Pevny L (2003). SOX2 functions to maintain neural progenitor identity. Neuron.

[16] Guedj F, Pennings JL, Massingham LJ, Wick HC, Siegel AE, Tantravahi U, Bianchi DW (2016). An integrated human/murine transcriptome and pathway approach to identify prenatal treatments for down syndrome. Sci Rep.

[17] Guidi S, Bonasoni P, Ceccarelli C, Santini D, Gualtieri F, Ciani E, Bartesaghi R (2008). Neurogenesis impairment and increased cell death reduce total neuron number in the hippocampal region of fetuses with Down syndrome. Brain Pathol.

[18] Guidi S, Giacomini A, Stagni F, Emili M, Uguagliati B, Bonasoni MP, Bartesaghi R (2018). Abnormal development of the inferior temporal region in fetuses with Down syndrome. Brain Pathol.

[19] Guihard-Costa AM, Khung S, Delbecque K, Menez F, Delezoide AL (2006). Biometry of face and brain in fetuses with trisomy 21. Pediatr Res.

[20] Hagey DW, Muhr J (2014). Sox2 acts in a dose-dependent fashion to regulate proliferation of cortical progenitors. Cell Rep.

[21] Hansen DV, Lui JH, Parker PR, Kriegstein AR (2010). Neurogenic radial glia in the outer subventricular zone of human neocortex. Nature.

[22] Howard BM, Zhicheng M, Filipovic R, Moore AR, Antic SD, Zecevic N (2008). Radial glia cells in the developing human brain. Neuroscientist.

[23] Huang H, Jeon T, Sedmak G, Pletikos M, Vasung L, Xu X, Yarowsky P, Richards LJ, Kostovic I, Sestan N (2013). Coupling diffusion imaging with histological and gene expression analysis to examine the dynamics of cortical areas across the fetal period of human brain development. Cereb Cortex.

[24] Hutton SR, Pevny LH (2011). SOX2 expression levels distinguish between neural progenitor populations of the developing dorsal telencephalon. Dev Biol.

[25] Jovanov Milosevic N, Judas M, Aronica E, Kostovic I (2014). Neural ECM in laminar organization and connectivity development in healthy and diseased human brain. Prog Brain Res.

[26] Kadhim HJ, Gadisseux JF, Evrard P (1988). Topographical and cytological evolution of the glial phase during prenatal development of the human brain: histochemical and electron microscopic study. J Neuropathol Exp Neurol.

[27] Kalebic N, Gilardi C, Stepien B, Wilsch-Brauninger M, Long KR, Namba T, Florio M, Langen B, Lombardot B, Shevchenko A (2019). Neocortical expansion due to increased proliferation of basal progenitors is linked to changes in their morphology. Cell Stem Cell.

[28] Karmiloff-Smith A, Al-Janabi T, D’Souza H, Groet J, Massand E, Mok K, Startin C, Fisher E, Hardy J, Nizetic D (2016). The importance of understanding individual differences in Down syndrome. F1000Research.

[29] Khan S, Vasung L, Marami B, Rollins CK, Afacan O, Ortinau CM, Yang E, Warfield SK, Gholipour A (2019). Fetal brain growth portrayed by a spatiotemporal diffusion tensor MRI atlas computed from in utero images. NeuroImage.

[30] Kida E, Wierzba-Bobrowicz T, Palminiello S, Kaur K, Jarzabek K, Walus M, Albertini G, Golabek AA (2010). Molecular chaperone alphaB-crystallin is expressed in the human fetal telencephalon at midgestation by a subset of progenitor cells. J Neuropathol Exp Neurol.

[31] Kostovic I, Sedmak G, Judas M (2019). Neural histology and neurogenesis of the human fetal and infant brain. NeuroImage.

[32] Larsen KB, Laursen H, Graem N, Samuelsen GB, Bogdanovic N, Pakkenberg B (2008). Reduced cell number in the neocortical part of the human fetal brain in Down syndrome. Ann Anat.

[33] Lee NR, Adeyemi EI, Lin A, Clasen LS, Lalonde FM, Condon E, Driver DI, Shaw P, Gogtay N, Raznahan A (2016). Dissociations in cortical morphometry in youth with down syndrome: evidence for reduced surface area but increased thickness. Cereb Cortex.

[34] Levman J, MacDonald A, Baumer N, MacDonald P, Stewart N, Lim A, Cogger L, Shiohama T, Takahashi E (2019). Structural magnetic resonance imaging demonstrates abnormal cortical thickness in Down syndrome: newborns to young adults. NeuroImage Clin.

[35] Lewitus E, Kelava I, Huttner WB (2013). Conical expansion of the outer subventricular zone and the role of neocortical folding in evolution and development. Front Hum Neurosci.

[36] Long KR, Newland B, Florio M, Kalebic N, Langen B, Kolterer A, Wimberger P, Huttner WB (2018). Extracellular matrix components HAPLN1, lumican, and collagen I cause hyaluronic acid-dependent folding of the developing human neocortex. Neuron.

[37] Lu J, Esposito G, Scuderi C, Steardo L, Delli-Bovi LC, Hecht JL, Dickinson BC, Chang CJ, Mori T, Sheen V (2011). S100B and APP promote a gliocentric shift and impaired neurogenesis in Down syndrome neural progenitors. PLoS ONE.

[38] Lu J, Lian G, Zhou H, Esposito G, Steardo L, Delli-Bovi LC, Hecht JL, Lu QR, Sheen V (2012). OLIG2 over-expression impairs proliferation of human Down syndrome neural progenitors. Hum Mol Genet.

[39] Lui JH, Hansen DV, Kriegstein AR (2011). Development and evolution of the human neocortex. Cell.

[40] Malik S, Vinukonda G, Vose LR, Diamond D, Bhimavarapu BB, Hu F, Zia MT, Hevner R, Zecevic N, Ballabh P (2013). Neurogenesis continues in the third trimester of pregnancy and is suppressed by premature birth. J Neurosci.

[41] Marin-Padilla M (1995). Prenatal development of fibrous (white matter), protoplasmic (gray matter), and layer I astrocytes in the human cerebral cortex: a Golgi study. J Comp Neurol.

[42] Miyazaki Y, Song JW, Takahashi E (2016). Asymmetry of radial and symmetry of tangential neuronal migration pathways in developing human fetal brains. Front Neuroanat.

[43] Nowakowski Tomasz J, Pollen Alex A, Sandoval-Espinosa C, Kriegstein Arnold R (2016). Transformation of the radial glia scaffold demarcates two stages of human cerebral cortex development. Neuron.

[44] Palminiello S, Jarzabek K, Kaur K, Walus M, Rabe A, Albertini G, Golabek AA, Kida E (2009). Upregulation of phosphorylated alphaB-crystallin in the brain of children and young adults with Down syndrome. Brain Res.

[45] Patkee PA, Baburamani AA, Kyriakopoulou V, Davidson A, Avini E, Dimitrova R, Allsop J, Hughes E, Kangas J, McAlonan G (2019). Early alterations in cortical and cerebellar regional brain growth in Down syndrome: an in vivo fetal and neonatal MRI assessment. NeuroImage Clin.

[46] Penisson M, Ladewig J, Belvindrah R, Francis F (2019). Genes and mechanisms involved in the generation and amplification of basal radial glial cells. Front Cell Neurosci.

[47] Pinson A, Namba T, Huttner WB (2019). Malformations of human neocortex in development—their progenitor cell basis and experimental model systems. Front Cell Neurosci.

[48] Pollen Alex A, Nowakowski Tomasz J, Chen J, Retallack H, Sandoval-Espinosa C, Nicholas Cory R, Shuga J, Liu Siyuan J, Oldham Michael C, Diaz A (2015). Molecular identity of human outer radial glia during cortical development. Cell.

[49] Rakic P (2003). Developmental and evolutionary adaptations of cortical radial glia. Cereb Cortex.

[50] Schmidt-Sidor B, Wisniewski KE, Shepard TH, Sersen EA (1990). Brain growth in Down syndrome subjects 15 to 22 weeks of gestational age and birth to 60 months. Clin Neuropathol.

[51] Smart IH, Dehay C, Giroud P, Berland M, Kennedy H (2002). Unique morphological features of the proliferative zones and postmitotic compartments of the neural epithelium giving rise to striate and extrastriate cortex in the monkey. Cereb Cortex.

[52] Subramanian L, Bershteyn M, Paredes MF, Kriegstein AR (2017). Dynamic behaviour of human neuroepithelial cells in the developing forebrain. Nat Commun.

[53] Supramaniam V, Vontell R, Srinivasan L, Wyatt-Ashmead J, Hagberg H, Rutherford M (2013). Microglia activation in the extremely preterm human brain. Pediatr Res.

[54] Takashima S, Becker LE, Armstrong DL, Chan F (1981). Abnormal neuronal development in the visual cortex of the human fetus and infant with down’s syndrome. A quantitative and qualitative Golgi study. Brain Res.

[55] Tarui T, Im K, Madan N, Madankumar R, Skotko BG, Schwartz A, Sharr C, Ralston SJ, Kitano R, Akiyama S (2019). Quantitative MRI analyses of regional brain growth in living fetuses with Down syndrome. Cereb Cortex.

[56] Thomsen ER, Mich JK, Yao Z, Hodge RD, Doyle AM, Jang S, Shehata SI, Nelson AM, Shapovalova NV, Levi BP (2016). Fixed single-cell transcriptomic characterization of human radial glial diversity. Nat Meth.

[57] Tournier JD, Calamante F, Connelly A (2010) Improved probabilistic streamlines tractography by 2nd order integration over fibre orientation distributions. In: Proceedings of the 18th annual meeting of ISMRM, City, pp 1670

[58] Tournier JD, Calamante F, Connelly A (2007). Robust determination of the fibre orientation distribution in diffusion MRI: non-negativity constrained super-resolved spherical deconvolution. NeuroImage.

[59] Tournier JD, Smith R, Raffelt D, Tabbara R, Dhollander T, Pietsch M, Christiaens D, Jeurissen B, Yeh CH, Connelly A (2019). MRtrix3: a fast, flexible and open software framework for medical image processing and visualisation. NeuroImage.

[60] Vasung L, Raguz M, Kostovic I, Takahashi E (2017). Spatiotemporal relationship of brain pathways during human fetal development using high-angular resolution diffusion MR imaging and histology. Front Neurosci.

[61] Vontell R, Supramaniam V, Thornton C, Wyatt-Ashmead J, Mallard C, Gressens P, Rutherford M, Hagberg H (2013). Toll-like receptor 3 expression in glia and neurons alters in response to white matter injury in preterm infants. Dev Neurosci.

[62] Vontell R, Supramaniam VG, Davidson A, Thornton C, Marnerides A, Holder-Espinasse M, Lillis S, Yau S, Jansson M, Hagberg HE (2019). Post-mortem characterisation of a case with an ACTG1 variant, agenesis of the corpus callosum and neuronal heterotopia. Front Physiol.

[63] Wisniewski KE (1990). Down syndrome children often have brain with maturation delay, retardation of growth, and cortical dysgenesis. Am J Med Genet Suppl.

[64] Wisniewski KE, Laure-Kamionowska M, Wisniewski HM (1984). Evidence of arrest of neurogenesis and synaptogenesis in brains of patients with Down’s syndrome. N Engl J Med.

[65] Xu G, Takahashi E, Folkerth RD, Haynes RL, Volpe JJ, Grant PE, Kinney HC (2014). Radial coherence of diffusion tractography in the cerebral white matter of the human fetus: neuroanatomic insights. Cereb Cortex.

[66] Zdaniuk G, Wierzba-Bobrowicz T, Szpak GM, Stepien T (2011). Astroglia disturbances during development of the central nervous system in fetuses with Down’s syndrome. Folia Neuropathol.

[67] Zecevic N (2004). Specific characteristic of radial glia in the human fetal telencephalon. Glia.

[68] Zecevic N, Chen Y, Filipovic R (2005). Contributions of cortical subventricular zone to the development of the human cerebral cortex. J Comp Neurol.

