# Supplementary figures and images for: Assessment of radial glia in the frontal lobe of fetuses with Down syndrome
Source: Acta Neuropathol Commun. 2020 Aug 20;8:141. doi: 10.1186/s40478-020-01015-3 (PMC7441567; doi:10.1186/s40478-020-01015-3)

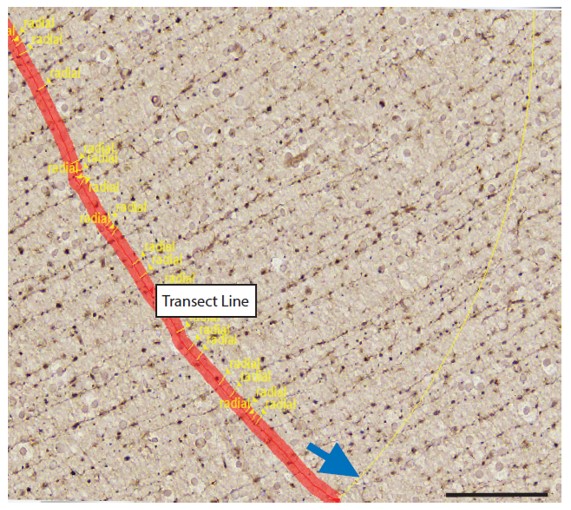

Supplement: Supplementary file 1 — Additional file 2: Figure S1. Schematic of Otolith Application. To measure the distance between radial glial fibres the Otolith Application was used. In each of the images assessed, three 1000 µm transect lines (red line) were drawn at different points on the edge of each scan. The outer reference circle was formed at the endpoint of each transect line (blue arrow). The application identified the circuli or radial structures (µm). The number of circulii and the distances between each radial projection was calculated and this data was analysed. Scale bar = 30 µm. [file 40478_2020_1015_MOESM1_ESM.jpg]

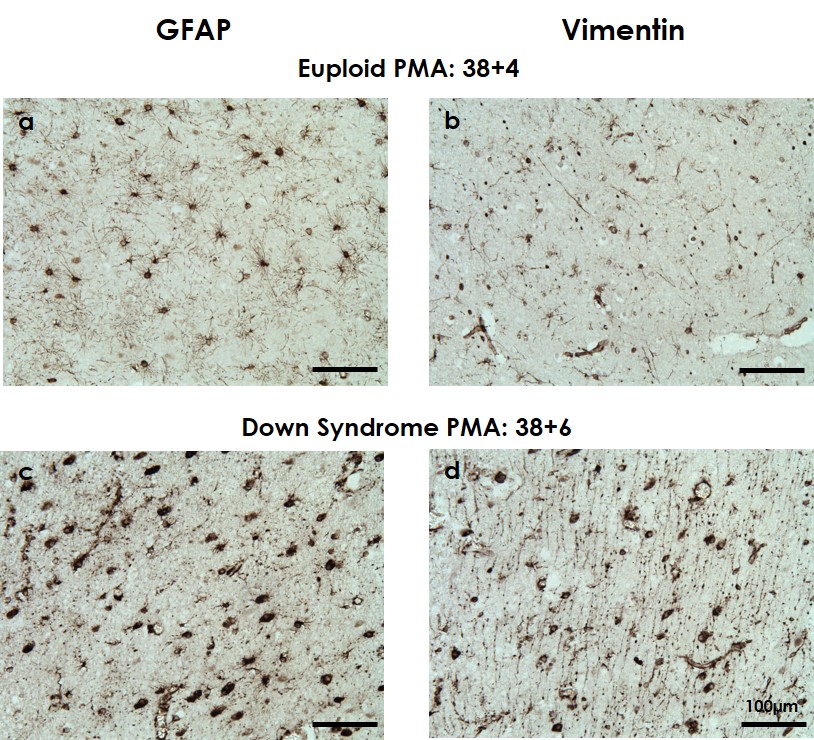

Supplement: Supplementary file 2 — Additional file 2: Figure S2. GFAP and Vimentin in late gestation. (A, C) GFAP and (B, D) Vimentin in the late gestation brain in (A, B) euploid age-matched (38+4 weeks PMA) and (C, D) DS (38+6 weeks PMA). DS case had Hypoxic-Ischemic Encephalopathy, with evidence of reactive astrocytes (C). Scale bar indicate 100 µm. [file 40478_2020_1015_MOESM2_ESM.jpg]
